# Predicting Progression-Free Survival Using MRI-Based Radiomics for Patients With Nonmetastatic Nasopharyngeal Carcinoma

**DOI:** 10.3389/fonc.2020.00618

**Published:** 2020-05-12

**Authors:** Hesong Shen, Yu Wang, Daihong Liu, Rongfei Lv, Yuanying Huang, Chao Peng, Shixi Jiang, Ying Wang, Yongpeng He, Xiaosong Lan, Hong Huang, Jianqing Sun, Jiuquan Zhang

**Affiliations:** ^1^Department of Radiology, Chongqing University Cancer Hospital and Chongqing Cancer Institute and Chongqing Cancer Hospital, Chongqing, China; ^2^Key Laboratory for Biorheological Science and Technology of Ministry of Education (Chongqing University), Chongqing University Cancer Hospital and Chongqing Cancer Institute and Chongqing Cancer Hospital, Chongqing, China; ^3^Key Laboratory of Optoelectronic Technology and Systems of the Education Ministry of China, Chongqing University, Chongqing, China; ^4^Department of Oncology and Hematology, Chongqing General Hospital, Chongqing, China; ^5^Department of Radiotherapy, Chongqing University Cancer Hospital and Chongqing Cancer Institute and Chongqing Cancer Hospital, Chongqing, China; ^6^Chongqing Key Laboratory of Translational Research for Cancer Metastasis and Individualized Treatment, Chongqing University Cancer Hospital and Chongqing Cancer Institute and Chongqing Cancer Hospital, Chongqing, China; ^7^Clinical Science, Philips Healthcare, Shanghai, China

**Keywords:** radiomics, prediction, progression-free survival, nasopharyngeal carcinoma, magnetic resonance imaging

## Abstract

**Objectives:** This study aimed to explore the predictive value of MRI-based radiomic model for progression-free survival (PFS) in nonmetastatic nasopharyngeal carcinoma (NPC).

**Methods:** A total of 327 nonmetastatic NPC patients [training cohort (*n* = 230) and validation cohort (*n* = 97)] were enrolled. The clinical and MRI data were collected. The least absolute shrinkage selection operator (LASSO) and recursive feature elimination (RFE) were used to select radiomic features. Five models [Model 1: clinical data, Model 2: overall stage, Model 3: radiomics, Model 4: radiomics + overall stage, Model 5: radiomics + overall stage + Epstein–Barr virus (EBV) DNA] were constructed. The prognostic performances of these models were evaluated by Harrell's concordance index (C-index). The Kaplan–Meier method was applied for the survival analysis.

**Results**: Model 5 incorporating radiomics, overall stage, and EBV DNA yielded the highest C-indices for predicting PFS in comparison with Model 1, Model 2, Model 3, and Model 4 (training cohorts: 0.805 vs. 0.766 vs. 0.749 vs. 0.641 vs. 0.563, validation cohorts: 0.874 vs. 0.839 vs. 836 vs. 0.689 vs. 0.456). The survival curve showed that the high-risk group yielded a lower PFS than the low-risk group.

**Conclusions:** The model incorporating radiomics, overall stage, and EBV DNA showed better performance for predicting PFS in nonmetastatic NPC patients.

## Introduction

Nasopharyngeal carcinoma (NPC) has obvious geographical distribution characteristics, especially in the south of China (1). Unfortunately, only 72.9% of patients with locoregionally advanced NPC have a 2-year progression-free survival (PFS) (2). Therefore, it is very important to improve the outcome of those patients. Individualized treatment based on precise stratification of PFS can improve the prognosis of NPC patients. This has led to a search for PFS prognostic factors, including clinical and image biomarkers.

To date, the American Joint Committee on Cancer/Union for International Cancer Control TNM staging system has been widely used to predict PFS for patients with NPC. Only the anatomy information including tumor size, lymph node status, and metastasis status were reflected by the TNM staging system (3). However, the latest eighth edition failed to accurately differentiate the PFS of stage II and III NPCs (4).

Plasma Epstein–Barr virus (EBV) DNA before treatment reflected the spread of tumor, and it has been used as a prognostic marker for PFS in patients with NPC (5–7). However, the accuracy of plasma EBV DNA is affected by a number of factors including DNA extraction, purification and stabilization methods, instruments, primers, and probes (8, 9).

Radiomics characterizes tissue heterogeneity by extracting and analyzing a large number of advanced CT, MRI, and PET imaging features (10). The radiomic features extracted from medical images can be used to predict the prognosis of tumors (11–14). In terms of NPC, a recent study showed that MR radiomics can significantly improve the efficacy of traditional TNM staging and clinical data in predicting PFS of patients with advanced NPC (15). Since the NPC is an EBV-related disease, the EBV DNA status is also taken into consideration in a study to develop multidimensional nomogram for predicting PFS in patients with advanced NPC (2). These studies indicated that MRI-based radiomic features and clinical characteristics may be valuable factors for predicting the PFS in advanced NPC patients. However, the MRI-based radiomic features including low-stage NPC patients for predicting the PFS were unknown.

Therefore, in the present study, we developed and validated MRI-based radiomics for predicting PFS in nonmetastatic NPC (stages I–IV_A_). In addition, we explored the predictive performance of the MRI-based radiomics in patients with different pretreatment EBV DNA levels.

## Materials and Methods

### Patients

Ethical approval and written informed consent were obtained for this retrospective study.

A total of 327 consecutive nonmetastatic NPC patients at our institute were enrolled between June 2013 and June 2017. The inclusion criteria were as follows: (a) pathologically confirmed NPC; (b) no treatments before registration; (c) no MR examination contraindications; (d) no distant metastasis; (e) no other primary tumor; and (f) complete clinical and MRI data.

All patients (*n* = 327) were randomly assigned to a training cohort (*n* = 230) and a validation cohort (*n* = 97). The demographic and clinical data (age, sex, hemoglobin level, and platelet count) were collected. Tumor was restaged according to the 8th Edition American Joint Committee on Cancer TNM Staging System.

In general, patients with stage I tumors were treated with curative radiotherapy (RT) alone, while those with stage II–IVA tumors were treated with radical concurrent chemoradiotherapy (CCRT), with/without induction chemotherapy (IC) or adjuvant chemotherapy (AC). The CCRT consisted of cisplatin for three to six cycles. IC and AC were cisplatin-based regimens every 3 weeks for 2–4 cycles. Patients who could not tolerate or refused chemotherapy did not receive chemotherapy. All patients were treated with intensity-modulated RT (30–33 fractions with five daily fractions per week for 6–7 weeks), and the total radiation doses were 60–72.6 Gy.

### Plasma EBV DNA

Plasma EBV DNA concentrations before treatment were routinely measured using a commercial extraction kit (Shanghai ZJ Bio-Tech Co. Ltd, China). The real-time polymerase chain reaction (PCR) analysis was performed on the Hongshi SLAN-9GP Real-Time PCR system. All tests were performed using our usual standard operating procedures according to the reagent operation instructions.

Patients with EBV DNA values of ≥500 copies/ml were assigned to the EBV DNA (+) group, and patients with EBV DNA values of <500 copies/ml were assigned to the EBV DNA (–) group.

### MRI Acquisition

All the patients were examined on a 1.5-T MRI scanner (Achieva, Philips Healthcare). T2-weighted (T2-w), T1-weighted (T1-w), and contrast-enhanced T1-weighted (CET1-w) MR images were acquired. The acquisition parameters were as follows: axial T2-w spin-echo images (FSE, TR = 5,013 ms, and TE = 100 ms), axial T1-w spin-echo images (FSE, TR = 456 ms, and TE = 15 ms), and axial CET1-w spin-echo images (FSE, TR = 450 ms, and TE = 15 ms). The slice thickness and interslice gap were 5 and 0.5 mm, respectively.

### Lesion Segmentation and Reproducibility Evaluation

Feature selection used the T2-w and CET1-w MR images. Regions of interest (ROIs) were first manually drawn slice by slice by one radiologist (observer 1) with 10 years of experience in head-and-neck MRI interpretation by using the in-house software developed by Philips. The ROIs covered the whole tumor.

We selected randomly 50 patients for reproducibility evaluation, and then we selected 100 radiomic features from each selected patient (50 features were randomly selected from T2-w-based radiomic features, and 50 features were randomly selected from CET1 image-based radiomic features) to evaluate the interobserver and intraobserver ICC. ROI segmentation was analyzed 1 month later by the same (observer 1) and one other radiologist (observer 2, 15 years of experience in head-and-neck MRI interpretation). The differences between the features extracted by observer 1 (first time) and those by observer 2, as well as the twice-extracted features by observer 1, were assessed by an independent Kruskal–Wallis *H* test.

### Radiomic Signature Building

Radiomic features were extracted from T2-w and CET1-w images using the Philips Radiomics Tool.

For feature selection, removing features with low variance was first performed to identify the significant features with threshold <0.01, and the least absolute shrinkage selection operator (LASSO) was explored to reduce the dimensionality of features. After that, the recursive feature elimination (RFE) based on support vector machine (SVM) was used to further choose the most valuable predictive features from the above features, and the dimensionality of features was further reduced. Finally, 20 key features were selected in the training cohort. After feature selection, a Cox model was built and used to construct a radiomic score (Rad-score).

### Prognostic Validation of the Radiomics

The potential correlations between PFS and radiomics were first assessed in the training group and then confirmed in the validation group. Kaplan–Meier survival was performed in both groups. The patients were assigned to high-risk and low-risk groups based on median Rad-score. Stratified analysis was used to determine the PFS in various subgroups, and high-risk and low-risk patients were compared. The C-index of the radiomics based on the T2-w and CET1-w images were calculated by the univariate Cox proportional hazards models.

The C-index was used to evaluate the prognostic performance of the five models (Model 1: clinical data, Model 2: radiomics, Model 3: overall stage, Model 4: radiomics + overall stage, Model 5: radiomics + overall stage + EBV DNA).

### Clinical Endpoints and Follow-Up

All patients were followed up every 3 months in the first 2 years, 6 months in years 3–5, and 12 months thereafter or until death. The follow-up time was defined from therapy initiation to the day of last examination or death. We set PFS as the endpoint (16, 17). PFS was defined from the 1st day of treatment to the date of disease progression (local recurrence or distant metastasis), death for any cause, or the last follow-up (censored). Disease progression was confirmed by biopsy pathology and/or imaging methods such as CT, MRI, or PET-CT (15).

### Statistical Analysis

The Mann–Whitney *U*-test was used to analyze quantitative variables of the training group and the validation group, and the *chi-square* test was used to analyze the qualitative variables. Those differences of the C-indexes in five models were compared by one-way ANOVA. Bonferroni *post-hoc* test was used for comparing the differences of the C-indexes between any two models. The statistical analysis was performed with SPSS software version 22.0 (SPSS IBM). LASSO in the “glmnet” package of R(version 3.6.2) and RFE based on SVR in Python(3.7) were used to select radiomic features to fit the Cox proportion model. The Kaplan–Meier survival and Cox proportional hazards regression analyses were respectively performed with the “survival” package and “rms” package in R (version 3.6.2). In Bonferroni *post-hoc* test, a two-sided *P* < 0.05/5 was considered statistically significant, and in all other tests, a two-sided *P* < 0.05 was considered statistically significant.

## Results

### Clinical Data

The clinical data of all patients are summarized in Table 1. There were no significant differences between the training and validation cohorts in clinical characteristics (*P* = 0.298–0.985). The median follow-up time was 38 months (range, 24–72 months), and the median PFS was 24 months (range, 3–60 months); 84 patients had progression (14 recurrences and 70 metastases), and 19 patients had died by the last follow-up.

**Table 1:** Characteristics of the patients in the training and validation cohorts.

	**Training cohort (*N* = 230)**	**Validation cohort (*N* = 97)**	***P***
**Gender**			0.645
Male	165 (71.7%)	72 (74.2%)	
Female	65 (28.3%)	25 (25.8%)	
**Age (years)**			
Median (IQR)	52.00 (45.00 ± 61.00)	52.00 (45.5 ± 60.5)	0.811
**Overall stage**			0.902
I	1 (0.4%)	0 (0.0%)	
II	32 (13.9%)	15 (15.5%)	
III	120 (52.2%)	48 (49.5%)	
IV_A_	77 (33.5%)	34 (35.0%)	
**T stage**			0.954
T1	20 (8.7%)	7 (7.2%)	
T2	100 (43.4%)	42 (43.3%)	
T3	65 (28.3%)	27 (27.9%)	
T4	45 (19.6%)	21 (21.6%)	
**N stage**			0.985
N0	18 (7.8%)	7 (7.2%)	
N1	62 (27.0%)	25 (25.8%)	
N2	111 (48.2%)	49 (50.5%)	
N3	39 (17.0%)	16 (15.5%)	
**Pretreatment EBV DNA**			
0	161 (70.0%)	67 (69.1%)	0.868
1	69 (30.0%)	30 (30.9%)	
**Pretreatment HB**			
Median (IQR)	137.50 (126.00–149.00)	137.00 (127.50–147.50)	0.792
**Pretreatment PLT**			
Median (IQR)	199.00 (153.00–253.00)	190.00 (157.50–242.00)	0.298
**PFS (months)**			
Median (IQR)	24.00 (14.00–29.00)	24.00 (15.00–28.00)	0.948

*HB, hemoglobin; PLT, platelets; PFS, progression-free survival*.

### Radiomic Signature Building

Extracted from T2-w images and CET1-w images are 1,227 features, adding up to 2,454 features. Twenty radiomic features were selected using the Rad-score prognostic model (Table 2).

**Table 2:** Radiomic feature selection result.

**MRI series**	**Selected features (CET1-w + T2-w)**	***P***
CET1-w	ShapeBased_Elongation	0.001218
CET1-w	WaveletGLCM_wavelet.LLH_ClusterShade	0.006283
CET1-w	WaveletGLCM_wavelet.LHL_MCC	0.006448
CET1-w	WaveletGLSZMwavelet.HLL _SmallAreaLowGrayLevelEmphasi	0.006222
CET1-w	WaveletGLSZMwavelet.LLL _LargeAreaLowGrayLevelEmphasis	0.001258
CET1-w	SquareGLSZM_square _LowGrayLevelZoneEmphasis	0.001393
CET1-w	LogarithmGLCM_logarithm_Correlation	0.000069
CET1-w	LogarithmGLCM _logarithm_MaximumProbability	0.000262
T2-w	WaveletFirstOrder_wavelet.LLH_Energy	0.000816
T2-w	WaveletFirstOrder_wavelet.LHH_Maximum	0.000045
T2-w	WaveletFirstOrder_wavelet.LHH_Median	0.008018
T2-w	WaveletFirstOrder_wavelet.HLL_Maximum	0.001324
T2-w	WaveletFirstOrder_wavelet.HLH_Median	0.000632
T2-w	WaveletGLCM_wavelet.HLH_ClusterShade	0.002597
T2-w	WaveletGLCM_wavelet.HHH_MCC	0.000009
T2-w	WaveletGLCM_wavelet.LLL _ClusterProminence	0.009537
T2-w	WaveletNGTDM_wavelet.LHL_Contrast	0.000443
T2-w	SquareNGTDM_square_Strength	0.000088
T2-w	ExponentialGLSZM_exponential_ZoneEntropy	0.000441
T2-w	ExponentialNGTDM_exponential_Busyness	0.000263

*The P-value for each radiomic feature associated with outcome was calculated using Cox proportional hazards regression. CET1-w, contrast-enhanced T1-weighted; T2-w, T2-weighted*.

**Figure 1:**
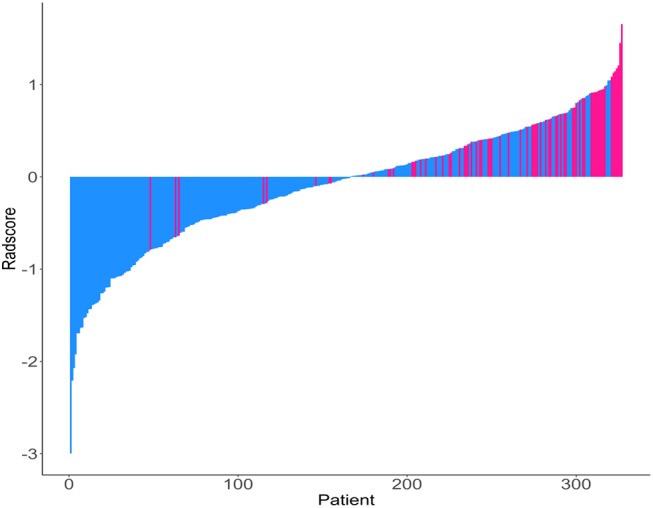
Rad-score for each patient. Dodger blue bars show scores for patients who survived without disease progression or were censored, while deep pink bars show scores for those who experienced progression or died.

The Rad-scores for each patient are shown in Figure 1. The Rad-scores between the training and validation cohorts were significantly different (*P* < 0.05).

### Reproducibility Evaluation of ROI Segmentation

There were no significant differences between the features of the two observers nor between first-extracted features and second-extracted features of observer 1's (*p*-values ranged from 0.576 to 0.784). The intraobserver ICC calculated based on observer 1's twice feature extraction ranged from 0.758 to 0.908, and the interobserver ICCs ranged from 0.752 to 0.889. Therefore, all outcomes were based on the features extracted by the observer.

### Prediction Performance of Models

The C-index of each model was shown in Table 3. The model of clinical data gained the lowest C-index in the training cohort [0.563 (95% CI: 0.493–0.634)] and validation cohort [0.456 (95% CI: 0.443–0.470)].

**Table 3:** C-index of the five models.

**Models**	**Training cohort (*N* = 230)**	**Validation cohort (*N* = 97)**
Clinical data	0.563 (95% CI: 0.493–0.634)	0.456 (95% CI: 0.443–0.470)
Overall stage	0.641 (95% CI: 0.604–0.679)	0.689 (95% CI: 0.677–0.701)
Radiomics	0.749 (95% CI: 0.713–0.783)	0.836 (95% CI: 0.823–0.849)
Radiomics + overall stage	0.766 (95% CI: 0.729–0.804)	0.839 (95% CI: 0.827–0.853)
Radiomics + overall stage + EBV DNA	0.805 (95% CI: 0.768–0.841)	0.874 (95% CI: 0.861–0.887)

The C-index of radiomics was higher than that of the overall stage in the training cohort [0.749 (95% CI: 0.713–0.783) vs. 0.641 (95% CI: 0.604–0.679)] and validation cohort [0.836 (95% CI: 0.823–0.849) vs. 0.689 (95% CI: 0.677–0.701)].

**Figure 2:**
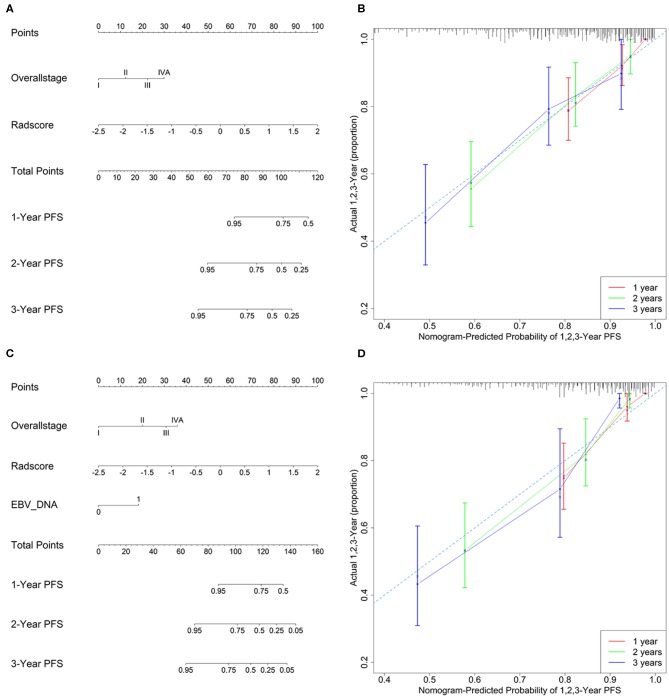
**(A)** A radiomic nomogram without EBV DNA integrating the radiomic signature with the TNM staging system. **(B)** Calibration curve of the radiomic nomogram without EBV DNA. **(C)** A radiomic nomogram with EBV DNA integrating the radiomic signature, TNM staging system, and EBV DNA. **(D)** Calibration curve of the radiomic nomogram with EBV DNA.

Model 4 integrating radiomics and overall stage gained a C-index of 0.766 (95% CI: 0.729–0.804) in the training cohort and 0.839 (95% CI: 0.827–0.853) in the validation cohort, which were higher than those of radiomics or overall stage alone. This nomogram without EBV DNA was shown in Figure 2A. The nomogram also showed good calibration (Figure 2B).

The number of EBV DNA (+) patients and EBV DNA (−) patients were 228 and 99, respectively. Model 5 integrating radiomics, overall stage, and EBV DNA gained the highest C-index of 0.805 (95% CI: 0.768–0.841) in the training cohort and 0.874 (95% CI: 0.861–0.887) in the validation cohort. The nomogram with EBV DNA and calibration curve integrating the three factors was shown in Figures 2C,D.

For low-stage NPC patients (*n* = 48), those C-indices of clinical data, radiomics, and EBV DNA were 0.533 (95% CI: 0.687–0.517), 0.759 (95% CI: 0.718–0.814), and 0.687 (95% CI: 0.633–0.718), respectively. The combined model (radiomics combined with EBV DNA) improved the predictive performance of PFS, its C-index was 0.772 (95% CI: 0.718–0.827).

### Kaplan–Meier Survival Analysis

**Figure 3:**
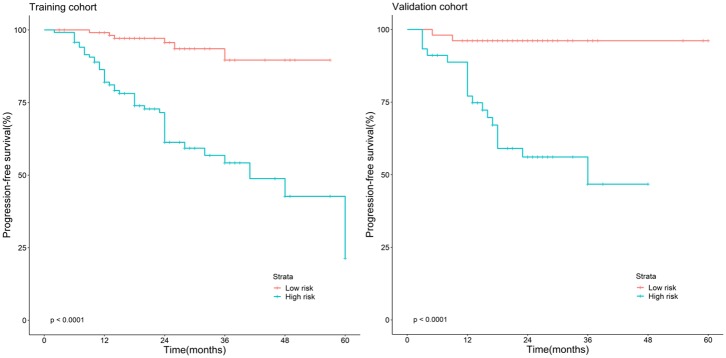
Stratified analyses were performed to estimate progression-free survival in the training cohort and validation cohort; high-risk patients show lower progression-free survival rate than low-risk patients, *P* < 0.05.

The Kaplan–Meier survival curves (Figure 3) were drawn based on the model of the radiomics combined with the overall stage. In the training cohort and validation cohort, the high-risk group yielded a lower PFS than the low-risk group (*P* < 0.05).

## Discussion

In our study, we developed an optimal model based on radiomics, overall stage, and EBV DNA to predict PFS in patients with stage I–IV_A_ NPC. The nomogram with EBV DNA improved the prediction performance of the nomogram without EBV DNA. We used the Rad-scores derived from the optimal model to stratify patients into high- and low-risk groups

The radiomics successfully translates medical imaging into mineable, quantitative, and high-dimensional imaging features, which offers an easy, effective, and reliable method of stratifying patients into risk groups and aids in decision making (18, 19). The radiomics may be an effective approach to predict PFS in patients with NPC via visualizing and quantifying intratumor heterogeneity. The study showed that the predictive performance of radiomics for PFS was higher than that for the traditional TNM overall stage, and the predictive performance of radiomics combined with overall stage was higher than that of radiomics or overall stage alone, which are consistent with previous studies (2, 15, 20).

The C-index of the radiomics combined with the overall stage in the study was slightly lower than that reported by the previous studies on advanced or T4 NPC (15, 21). The possible explanation may be that the present study included 327 patients with T1–T4 NPC, which may improve the generalizability of the prediction models.

Recent evidence indicates that plasma EBV DNA may not only reflect tumor burden but also be an index of other tumor features, such as accessibility to angiogenesis, circulation, metabolic activity, tumor cell kinetics, and metastatic potential (22). Moreover, several studies demonstrated that pretreatment EBV DNA is relevant to PFS of patients with NPC (9, 23). On the basis of the above studies, we performed two nomograms with or without pretreatment EBV DNA on NPC patients. Our result indicates that the nomograms with pretreatment EBV DNA improved the prediction performance of those without pretreatment EBV DNA; this finding with a larger sample size was consistent with a previous one (2).

The Kaplan–Meier survival curves based on the radiomics combined with overall stage model can commendably stratify the PFS of NPC patients, which may contribute to the accurate stratification of patients for individual treatment strategies in clinical practice and the improvement of the clinical outcomes of patients with NPC. The PFS of the high-risk group was lower than that of the low-risk group, which was similar to previous studies (18, 20, 21, 24), and it is helpful for administering individualized treatment plans.

Our study has some limitations. First, this study was conducted in a single center. The results should be interpreted cautiously and verified by a large-sample-size, multicenter study. Second, the mean follow-up time was relatively short; a longer follow-up time is required to predict the 5-year PFS rates. Third, radiomics always attempts to find the most valuable feature in various data, while we only analyze T2-w and CET1-w images. The analysis of multiparameter data may help improve the quality of the model.

In conclusion, our study showed that the model incorporating radiomics, overall stage, and EBV DNA showed optimal performance for predicting PFS in nonmetastatic NPC patients. The combined model can stratify patients into low- and high-risk groups; it may provide additional information to personalized treatment decision in nonmetastatic NPC.

## Data Availability Statement

The datasets generated for this study are available on request to the corresponding author.

## Ethics Statement

The studies involving human participants were reviewed and approved by the ethics committee of Chongqing University Cancer Hospital. The patients provided written informed consent to participate in this study.

## Author Contributions

HS, YuW, and DL: conception and design. JZ, YiW, and HH: administrative support. RL, YyH, CP, and YpH: provision of study materials or patients. XL and SJ: data collection and collation. XL and JS: data processing and analysis. All authors manuscript writing and final approval of manuscript.

## Conflict of Interest

JS was employed by the company Philips Healthcare. The remaining authors declare that the research was conducted in the absence of any commercial or financial relationships that could be construed as a potential conflict of interest.

## Abbreviations

EBV: Epstein–Barr virus
PFS: Progression-free survival
LASSO: Least absolute shrinkage and selection operator
PCR: Polymerase chain reaction
CE-T1WI: Contrast-enhanced T1-weighted image
T2WI: T2-weighted image
CI: Confidence interval
NPC: Nasopharyngeal carcinoma.
